# Book News

**Published:** 1902-08

**Authors:** 


### BOOK NEWS.

Every veterinarian of Pennsylvania should obtain through his Representative a copy of *Part I., of the Report of the Department of Agriculture* for 1901. It is replete with matters of great interest and importance to the profession of this country, and especially to Pennsylvania's. Among some of the matters recorded and discussed are the Act for Prevention of Spreading of Diseases from Carcasses of Diseased Animals; the Period of Gestation of Domestic Animals, Study and Experiments of Tubercle Bacillus, Anthrax and Black Quarter, Cattle Food and Composition of Feeding Stuffs, Analysis of Condimental Foods and Study of Cultures of Various Diseases Producing Micro-organisms.

This book also contains an article on the Division of Veterinary Science, with discussions; glanders, hog cholera, rabies and tuberculosis; remarks on the question of milk supplies, with statistics of the milk supplied, and the report of the State veterinarians.

